# Dementia in the United Arab Emirates: Factors Affecting the Time From Symptom Emergence to Formal Diagnosis

**DOI:** 10.1192/bjo.2024.174

**Published:** 2024-08-01

**Authors:** Syed Fahad Javaid, Zubaida Shebani, Gabriel Andrade

**Affiliations:** 1 Department of Psychiatry and Behavioral Sciences, College of Medicine and Health Sciences, United Arab Emirates University, Al Ain, UAE; 2 Sultan Qaboos University, Muscat, Oman; 3 Ajman University, Ajman, UAE

## Abstract

**Aims:**

Dementia is a debilitating neurodegenerative disorder that can negatively impact the lives of those affected and their families. Providing safe and person-centered care for individuals living with dementia is a global need with particular emphasis on providing individuals and their families with a rapid diagnosis of their condition following the commencement of symptoms. This study aimed to establish the mean duration of cognitive symptoms before a formal diagnosis of dementia is given in the United Arab Emirates (UAE). We also studied demographic and symptom-specific factors affecting the time for dementia to be formally diagnosed. Our study examined a global issue through a more localized lens to identify areas for improvement.

**Methods:**

The study involved extracting and analyzing anonymous data from the electronic medical records of dementia patients at Al-Ain Hospital, UAE. Following ethical approval, the data for individuals diagnosed with any form of dementia from 01/01/2010 to 31/12/2019 were extracted using a set of related diagnostic codes. A short questionnaire was completed for every record that matched the search criteria. Demographic information was collected in addition to details of diagnosis, presenting symptoms, comorbidities, and medications.

A two-tailed independent t-test was conducted to assess the effect of demographic characteristics (gender, nationality, and age) on the time to receive a diagnosis of dementia. A one-way ANOVA was conducted to assess the effect of initial symptoms, including forgetfulness, agitation/aggression, and hallucinations, on the time taken to receive a diagnosis.

**Results:**

Out of the total sample of 825, 442 (53.6%) were females, with 518 (63%) being Emirati citizens. The mean age of the studied sample at the time of diagnosis was 78 years (SD = 11.1). Alzheimer's dementia, 335 (40.6%), was the most common subtype diagnosed. The mean duration of symptoms (DUS) before formal diagnosis was 34.6 months (SD = 28.8). A statistically significant relationship was found between age and DUS, with those over 70 years of age at the time of diagnosis more likely to have a longer DUS (p < 0.001). There was a statistically significant mean difference in the DUS and some initial symptoms, namely agitation/aggression(p < 0.001), lability (p < 0.003), disinhibition (p < 0.001), and hallucinations (p < 0.001).

**Conclusion:**

To our knowledge, this is the first study of its kind in the UAE. Future investigation in this area is much needed, and this study will provide the foundations for dementia awareness campaigns encouraging early presentation to the services.

No financial sponsorship has been received for this study.

